# PREVALENCE OF SINGLE AND MULTIPLE CHRONIC CONDITIONS AMONG RESETTLED BHUTANESE OLDER ADULTS IN OHIO

**DOI:** 10.1093/geroni/igad104.2093

**Published:** 2023-12-21

**Authors:** Isha Karmacharya, Aman Shrestha, Janardan Subedi, Saruna Ghimire

**Affiliations:** Miami University, Oxford, Ohio, United States; Miami University, Oxford, Ohio, United States; Miami University, Oxford, Ohio, United States; Miami University, Oxford, Ohio, United States

## Abstract

**Background:**

Migrants and refugees are more prone to chronic health conditions, which can worsen with age and accumulated stressors. Bhutanese older adults with refugee backgrounds in the US have not been well-studied. This study aimed to assess the prevalence of chronic diseases (single and multiple conditions) and to identify factors associated with multimorbidity among resettled Bhutanese older adults in Ohio. Additionally, the study compared the prevalence of chronic diseases with state-level data for other racial/ethnic groups.

**Methods:**

Structured interview was conducted among 276 resettled Bhutanese, aged 55 years and above, in four major Ohio cities (Columbus, Akron, Cleveland, and Cincinnati), selected through snowball sampling with local Bhutanese organizations. State-level data of people, aged 55 years and above in Ohio, from the 2021 Behavioral Risk Factor Surveillance System for Non-Hispanic White, Non-Hispanic Black, Hispanic, and Asian populations were used for comparison. Binary logistic regression was used to identify factors associated with multimorbidity.

**Results:**

Only 14.1% of participants were free of chronic diseases, while 62.3% had multimorbidity. Depression (31.8%), diabetes (42.8%), and respiratory diseases (26.4%) in older Bhutanese were considerably higher than in other racial/ethnic groups. Hypertension prevalence (63.0%) was higher than all other race/ethnic groups, except Non-Hispanic Black individuals (72.3%). Poor self-reported health and the presence of depressive symptoms had higher odds of having multimorbidity. Study Implications: This study underscores valuable insight into the disease burden among resettled Bhutanese older adults and can inform stakeholders to develop targeted public health programs and practices to meet the needs of minority populations.

